# An integrated workflow for robust alignment and simplified quantitative analysis of NMR spectrometry data

**DOI:** 10.1186/1471-2105-12-405

**Published:** 2011-10-20

**Authors:** Trung N Vu, Dirk Valkenborg, Koen Smets, Kim A Verwaest, Roger Dommisse, Filip Lemière, Alain Verschoren, Bart Goethals, Kris Laukens

**Affiliations:** 1 Department of Mathematics and Computer Science, University of Antwerp, Antwerp, Belgium; 2 Vlaamse Instelling voor Technologisch Onderzoek (VITO), Mol, Belgium; 3 Department of Chemistry, University of Antwerp, Antwerp, Belgium; 4 Biomedical Informatics Research Center Antwerp (biomina), University of Antwerp, Antwerp, Belgium; 5 Interuniversity Institute for Biostatistics and Statistical Bioinformatics, Hasselt University, Diepenbeek, Belgium

## Abstract

**Background:**

Nuclear magnetic resonance spectroscopy (NMR) is a powerful technique to reveal and compare quantitative metabolic profiles of biological tissues. However, chemical and physical sample variations make the analysis of the data challenging, and typically require the application of a number of preprocessing steps prior to data interpretation. For example, noise reduction, normalization, baseline correction, peak picking, spectrum alignment and statistical analysis are indispensable components in any NMR analysis pipeline.

**Results:**

We introduce a novel suite of informatics tools for the quantitative analysis of NMR metabolomic profile data. The core of the processing cascade is a novel peak alignment algorithm, called hierarchical Cluster-based Peak Alignment (CluPA). The algorithm aligns a target spectrum to the reference spectrum in a top-down fashion by building a hierarchical cluster tree from peak lists of reference and target spectra and then dividing the spectra into smaller segments based on the most distant clusters of the tree. To reduce the computational time to estimate the spectral misalignment, the method makes use of Fast Fourier Transformation (FFT) cross-correlation. Since the method returns a high-quality alignment, we can propose a simple methodology to study the variability of the NMR spectra. For each aligned NMR data point the ratio of the between-group and within-group sum of squares (BW-ratio) is calculated to quantify the difference in variability between and within predefined groups of NMR spectra. This differential analysis is related to the calculation of the F-statistic or a one-way ANOVA, but without distributional assumptions. Statistical inference based on the BW-ratio is achieved by bootstrapping the null distribution from the experimental data.

**Conclusions:**

The workflow performance was evaluated using a previously published dataset. Correlation maps, spectral and grey scale plots show clear improvements in comparison to other methods, and the down-to-earth quantitative analysis works well for the CluPA-aligned spectra. The whole workflow is embedded into a modular and statistically sound framework that is implemented as an R package called "speaq" ("spectrum alignment and quantitation"), which is freely available from http://code.google.com/p/speaq/.

## Background

Nuclear magnetic resonance spectroscopy (NMR) is a powerful and widely applied analytical high-throughput technique to reveal and compare the quantitative metabolic profile of a given tissue in relation to various environmental and clinical parameters. A typical NMR spectrum is composed out of an x-axis, which indicates the resonance frequencies of the observed molecule, and a y-axis, which denotes the corresponding intensities, i.e., abundance. To analyse experimental NMR datasets, multivariate methods such as principle components analysis (PCA) or univariate techniques like Student-t test are commonly applied. However, chemical and physical sample variations due to, among others, differences in pH, temperature, ion content and the concentration of metabolites, make the analysis of the data challenging. To address these challenges, several preprocessing steps are commonly applied, including noise reduction, normalization, baseline correction, peak picking and spectrum alignment, prior to statistical analysis.

A crucial and often depreciated aspect in this process is peak alignment, which aims to compensate for small variations in the position of corresponding peaks between spectra. A number of spectral alignment approaches have previously been proposed. However, most of them come with particular disadvantages. For example, some methods use dynamic programming, like Correlation Optimized Warping (COW) and Dynamic Time Warping (DTW) [1,2]. Due to their computational complexity an alignment task based on these techniques may take hours. Several authors worked towards solutions to speed up this alignment process [3] used a Fast Fourier Transformation (FFT) cross-correlation engine to improve the alignment speed (PAFFT). They also introduced an advanced extension, called recursive peak alignment by FFT (RAFFT), which recursively divides the spectrum into meaningful segments and aligns them until a certain degree of goodness is obtained. Some advanced peak picking approaches are Recursive Segment-wise Peak Alignment (RSPA) [4] and Generalized Fuzzy Hought Transform (GFHT) [5]. Other authors applied search algorithms to peak alignment, such as genetic algorithms in PAGA [6] and beam searching in PABS [7]. Recently [8], introduced the interval-correlation-shifting (Icoshift) algorithm, which aligns spectra by maximizing the cross-correlation between user-defined intervals.

Another approach that is commonly employed for the peak alignment of mass spectral data is based on hierarchical clustering and could be applied as well on NMR spectral data [9-14]. Most of these methods apply hierarchical clustering to the entire collection of all peaks from the individual spectra and "cut off" the resulting dendrogram at a suitable height to produce a number of clusters used for alignment. This approach works well on NMR data that is already calibrated to some extent. However, in some datasets, the peak positions of chemical resonances are significantly shifted between the samples. This strong shift could make the NMR spectra unclear to separate, which may lead to the wrong clustering, i.e. alignment, of peaks. The effect of strongly shifted spectra also challenges the methods based on spectral binning, like COW and Icoshift, because peaks could mistakenly be assigned to the wrong bins.

To address the problems with misaligned spectra, we first focus on the development of a robust and highly confident alignment algorithm. The method is based on a peak-picking approach for NMR spectra, called hierarchical Cluster-based Peak Alignment (CluPA). The alignment is embedded in a workflow (called *speaq*: "spectrum alignment and quantitation") that starts with deriving the peak list of each spectrum separately, followed by selecting the reference spectrum to which other spectra are aligned.

The high-quality of the alignment introduced here allows for the use of standard and stable components up- and downstream the processing pipeline. For example, prior to the peak-picking, noise reduction is accomplished by wavelet filtering. Further more, differential regions in an NMR spectra between two conditions (control versus case) are determined based on the ratio of the between-group to within-group sums of squares (BW-ratio), as proposed by [15]. The BW-ratio is calculated for each data point in the NMR spectra. Because the spectra are well aligned, no variability, due to shifted peaks, is added to the BW-statistic.

## Implementation

**Figure 1:**
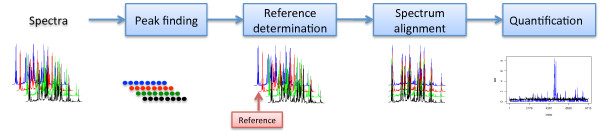
**Outline of the quantitative analysis suite**. An overview of all the steps of the workflow.

The complete workflow combines four major steps, illustrated in Figure 1 and itemized below. Each of these major steps is discussed in more detail in separate subsections. The core of the workflow, is a novel approach for spectral alignment.

1. Noise reduction and peak picking: remove instrument noise and collect peaks from the NMR spectra.

2. Reference determination: selecting a dominant and representative spectrum, which is used as template for the alignment.

3. Peak alignment: shifts are estimated by hierarchical clustering on the selected peaks and the template.

4. Quantification: determination of differential region in the NMR spectra based on the BW-ratio.

### Noise reduction and peak finding

Although peak detection is a common step in the analysis of spectral data, it remains a challenging task, due to the noise inherent to many spectrometric techniques. A typical peak detection approach consists of three steps: smoothing or denoising, baseline correction and the actual peak picking. For each step, several methods have been developed, but there does not seem to be a "golden standard". Since the detected peaks are the input of the alignment procedure, selecting an appropriate technique is important. In our implementation, the method of [16], recommended in [17] is chosen. It employs continuous wavelet transform (CWT) to detect peaks in a NMR spectrum. We used the package MassSpecWavelet [16] which integrates wavelet-based filters in the smoothing step, continuous wavelet transform for baseline correction, and signal to noise ratio thresholding and ridge lines for the peak picking [16,17].

In our approach, the method of Du et al. [16] could not be applied to the whole spectrum directly, due to computational complexity and possible insensitivity towards low intensity peaks in the NMR spectrum. To address this, the function peakdetectionCWT of the library MassSpecWavelet is used only on partitions of the spectrum. Therefore the spectrum was divided into predefined equal window-sized segments. Next, the function peakdetectionCWT is applied to a four-segment sized window that slides along the spectrum, one segment at a time. All detected peaks are subsequently merged into a final peaklist. This strategy avoids missing peaks in the margin of a segment. The segment size was chosen as a power of 2, in order to boost the speed of the CWT [16,17]. It should be noted that the segment size has to be wide enough to cover the widest peaks in spectra. Noisy peaks are removed by applying a baseline minimum intensity threshold (baselineThresh). The optimal threshold is dataset-dependent, and should be supplied a priori by the user. For the selection of suitable parameters for the peak detection, we refer to [16]. The user may also use alternative peak detection methods, but it is important to take into account that the quality of the alignment and the downstream analysis can suffer from counterfeit peak detection.

### Reference determination

The second step in the workflow consists of the selection of a reference spectrum. The reference spectrum serves as a kind of template for other spectra to be aligned against. The ideal reference contains the highest number of common chemical constituents [4]. Several methods exist to select an appropriate reference spectrum: either prior knowledge about the dataset can be used, or the reference can be selected according to a predefined criterion [4,18] propose a method based on the product of the Pearson correlation coefficients between spectra. The approach of [8] creates a virtual "average" spectrum as a reference. Alternatively, the spectrum that is most similar to the loading of the first principle component in a PCA model can be chosen [18].

It is difficult to define "common chemical constituents" when spectra are ill aligned. To ensure robust reference selection, we introduce an alternative method that incorporates a heuristic strategy to find the optimal template. It selects the spectrum with the largest goodness as the reference, which is defined by the sum of the inter-spectrum distances, according to formula 1 and 2:

(1) $$goodness\left(S\right)=-\underset{T}{\sum }\underset{pT\in T}{\sum }\underset{pS\in S}{\min}\left(\left|pS-pT\right|\right)$$

(2) $$ref=argmax_{S}\left(goodness\left(S\right)\right)$$

where *pS *and *pT *represent the resonance frequencies, i.e., location along the x-axis, of the corresponding peaks of spectrum *S *and *T*, respectively.

The distance is computed by the sum of the minimum distances between every peak *pT *of *T *and the peaks *pS *of *S*, because the closest peak is chosen as the best candidate to be matched with *pT*. Note, that the distance is not symmetric, that is the distance of *S *to *T *and the distance of *T *to *S *are not necessarily equal.

In principle, the method projects each high-dimensional NMR spectrum as a data-point in a one-dimensional space and selects the data-point in the middle of the data cloud as the reference. A possible disadvantage of this method is that it favours the spectrum with a high number of peaks, which is not necessarily in the center of the data cloud. If that is the case, a lower ranked spectrum could be selected instead. Ideally, the perfect reference candidate has enough peaks corresponding to chemical resonance areas, i.e. frequencies and the peaks of the reference stay at the center of the distribution of peaks of other spectra in order to minimize the distance.

The computational complexity to find the reference spectrum includes *O*(*n*) for finding the minimum value and the second sum in formula (1); and *O*(*m*) for the other sum of formula (1) and the maximum of formula (2); where *n *is the maximum number of peaks of a spectrum and *m *is the number of spectra. The total complexity is then *O*(*O*(*n*) * *O*(*n*) * *O*(*m*) + *O*(*m*)) or *O*(*n*^2^*m *+ *m*) or *O*(*n*^2^*m*). It should be noticed that good peak detection is required to restrain the computational complexity within acceptable limits. In practice, finding the reference spectrum is fast, since the number of peaks *n *of a spectrum is small, given that peak selection up-stream of the workflow is adequate.

### Spectrum alignment

The alignment method is called hierarchical Cluster-based Peak Alignment (CluPA), and is based on shifting segments of the original spectra, first globally and then in smaller local segments. In the first iteration, CluPA does the alignment on the whole spectrum. In subsequent iterations, CluPA recursively splits the segment(s) of previous steps into two smaller segments, and applies an individual alignment shift to each of these. The boundaries of these segments are defined by applying a hierarchical clustering algorithm on the combined peak list of the reference and the target spectrum. The clustering is based on the distance between peaks: peaks are hierarchically combined into a tree according to their distance. After each round, the segment is split into two smaller segments by cutting at the second level of the hierarchical tree. At each iteration, the present segments of the original spectra are aligned to the reference spectrum. To this end, an optimal shift step is determined between the target and reference spectrum within the present segments. This shift is subsequently applied to the corresponding segment in the target spectrum. The process is repeated until a segment contains only peaks of a single spectrum, or until there are only two peaks left in the list. A detailed outline of the algorithm is presented in the following pseudo-code.

### Input

• *targetSpec*: original spectrum to be aligned

• *refSpec*: original reference spectrum

• *mergedPeakList*: merged list of all peaks detected in targetSpec and refSpec

### Output

• *targetSpec*: spectrum after alignment

**CluPA**(*refSpec*, *targetSpec*, *mergedPeakList*)

1. startPoint, endPoint = **findShiftRegion**(*refSpec, targetSpec, mergedPeakList*)

2. shiftStep = **findShiftStep**(*refSpec, targetSpec, startPoint, endPoint*)

3. targetSpec, mergedPeakList = **shift**(*targetSpec, mergedPeakList, shiftStep, startPoint, endPoint*)

4. hClusterTree = **buildhCluster**(*mergedPeakList*)

5. leftPeakList = **cutLeftTree**(*hClusterTree*, 2)

6. if **satifiedCondition**(*leftPeakList*) then

7. targetSpec = **CluPA**(*refSpec, targetSpec,leftPeakList*)

8. rightPeakList = **cutRightTree**(*hClusterTree*, 2)

9. if **satifiedCondition**(*rightPeakList*) then

10. targetSpec = **CluPA**(*refSpec, targetSpec,rightPeakList*)

11. **return**(*targetSpec*)

Step 1: **findShiftRegion**(*refSpec, targetSpec, mergedPeakList*): determines the region of the spectra for alignment, which must contain all peaks of the *mergedPeakList*. The left most of the region is the lowest intensity point in the spectral region on the left side of the list. Similarly, the end point of the region is the lowest intensity point in the spectral region on the right side of the list. It takes linear time *O*(*d*) to select the lowest intensity point, where *d *is the length of the spectral region. This value rapidly decreases when the algorithm iterates with increasingly smaller regions.

Step 2: **findShiftStep**(*refSpec, targetSpec, startPoint, endPoint*): finds a shift step by using FFT Cross-Correlation. The step has a computational cost *O*(*dlogd*). In implementation, the function is simulated from the function FFTcorr in RAFFT [3]. Alternatives, for example Pearson correlation and Weighted Cross Correlation in [19] could also be applied as an alternative, however, Pearson correlation takes more time than using FFT cross correlation.

Step 3: **shift**(*targetSpec, mergedPeakList, shiftStep, startPoint, endPoint*): moves *shiftStep *- the number of points on the current region of *targetSpec *spectrum and peak list. This simple step takes linear time *O*(*d*) to complete.

Step 4: **buildhCluster**(*mergedPeakList*): creates a hierarchical cluster tree from the peaks of the *mergedPeakList*. In the first step, each cluster has a single peak. The hierarchical cluster tree is built by in turn grouping the closest two clusters together. There are three common ways to define the distance between two clusters: Single linkage, complete linkage and average linkage [20]. Single linkage distance of two clusters is the minimum distance of two objects or peaks in each cluster, complete linkage distance is computed by maximum distance of the two objects, and average linkage is calculated by the average of all distances between any pairs of objects between the two clusters. By default, the average linkage is selected as the distance for clustering, however, other types of distance could be applied. This step takes *O*(*n*^2^) to *O*(*n*^2^*logn*) [20], depending on which linkage distance is selected, where *n *is the number of elements in the peak list. The calculation of single linkage distance is faster than its alternatives. In the implementation, the standard function *hclust() *in the stats package of the R software (R version 2.11.1 (2010-05-31)) is applied.

Step 5: **cutLeftTree**(*hClusterTree*, 2): gets the peaks corresponding to the left tree at the second level of *hClusterTree*.

Step 8: **cutRightTree**(*hClusterTree*, 2): gets the peaks corresponding to the right tree at the second level of *hClusterTree*.

In the implementation, the function *cutree*() of the stats package is applied. It takes *O*(*n*) to be finished.

Step 6 and 9: **satifiedCondition**(*leftPeakList*) verifies whether the condition that determines whether a tree should be further divided are met. The tree has to contain peaks of both reference and target spectra, otherwise the spectral region does not need to be further aligned.

The maximum computational complexity in each stage is *O*(*O*(*d*) + *O*(*dlogd*) + *O*(*d*) + *O*(*n*^2^*logn*) + 2 * *O*(*n*)) or *O*(*dlogd *+ *n*^2^*logn*), and *O*(*logn*) for the recursive loop. Therefore, the total complexity of the algorithm is *O*(*logn*) * *O*(*dlogd *+ *n*^2^*logn*).

**Figure 2:**
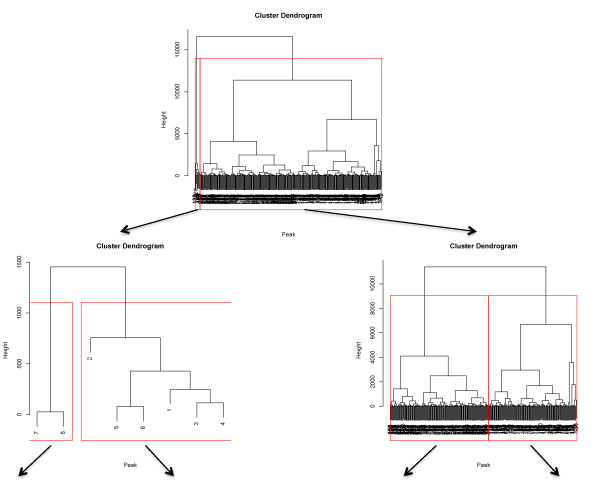
**Peak list segmentation by CluPA**. At each iteration, CluPA cuts the hierarchical tree into two subtrees (marked by red lines). This process is recursively iterated with each subtree until the stopping criteria are satisfied. The arrows point to the corresponding subtrees after the cut.

**Figure 3:**
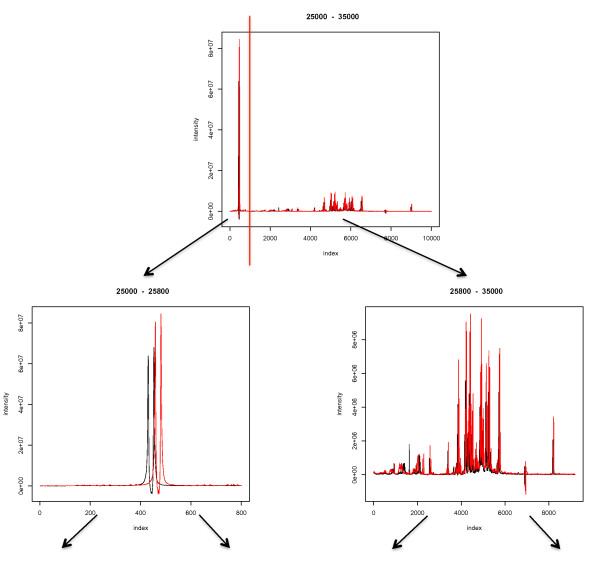
**Spectral view that corresponds to the hierarchical segmentation of Figure 2**. The spectra in range of 25000 to 35000 are divided (by the vertical red line) into two smaller ranges that will undergo further alignment. The arrows point to the corresponding spectra after the cut.

Figure 2 demonstrates how the algorithm selects peak groups from the merged peak list, by hierarchical clustering. The red lines are bounding the second-level children of the tree, which corresponds to the segments that will be individually processed. The trees shown in Figure 2 correspond to regions in the original spectrum, which are depicted in Figure 3, and will undergo individual alignment.

### Quantitative analysis

The thorough alignment of the NMR spectra, outlined in previous section, allows for a very straightforward and simple quantification analysis. The data returned by CluPA are well aligned and presented in a rectangular format. This means that the data are represented as a matrix in which the rows denote the spectra and the columns indicate the resonance frequency instances. The quantification strategy is inspired by the method proposed by Tan et al [21] for annotating regions of significance in SELDI-TOF spectra. In our approach, for each frequency instance in the matrix, we performed an analysis of the data based on the ratio of the between-group to within-group sums of squares (BW-ratio) as proposed by Dudoit et al [15]. This ratio is related to the F-statistic or one-way ANOVA, but we do not make assumptions about the distribution and the independency of the data. For example, the BW-ratio of frequency instance *j *is calculated as

(3) $$BW\left(j\right)=\frac{\Sigma_{i}\Sigma_{k}I\left(y_{i}=k\right){\left(x_{kj}^{-}-x_{.j}^{-}\right)}^{2}}{\Sigma_{i}\Sigma_{k}I\left(y_{i}=k\right){\left(x_{ij}-x_{kj}^{-}\right)}^{2}}$$

where $x_{.j}^{-}$ and $x_{kj}^{-}$ denote the average intensity level of frequency instance *j *between all NMR spectra and within the NMR spectra belonging to group *k *only, respectively. The variable *I *is an indicator for the group. The variable *x*_*ij *_denotes the *i*-th intensity values of frequency *j *belonging to group *k*. Statistical inference on the BW-statistic is based on the sampled null distribution from the observed data. For this purpose the average of the groups $x_{kj}^{-}$ is subtracted from the data. Next, data points are sampled for each frequency instance *j *using the same grouping as in the original setting. Based on the sampled data, the BW-ratio is calculated, which is representative for data showing no difference in the group means. This computation is iterated with replacement of the data until a well sampled null distribution is obtained. For each frequency *j*, the value of the BW-statistic is determined for the desired alpha-level based on the sampled null distribution.

We are aware that NMR-spectra are correlated between consecutive frequency instances and cannot be treated as independent variables. A model which takes into account such a correlation structure to discern noise peaks from bona fide molecule peaks is part of future research. In addition, testing for every possible data entry can make the detection of differential frequency instances inefficient, because a valid NMR-peak can be composed out of multiple, redundant, data points. Therefore, correction of multiplicity is done by using the conservative Bonferoni correction. Doing so, the alpha-level is adjusted for the average number of peaks returned by the peak picking algorithm used in the first step of the workflow. We can already announce that this ad-hoc quantitative analysis is very fast and robust.

An interesting point is that the differential frequencies do not necessarily represent the apex of the NMR-peak, but are rather found in the shoulder of the peak. We also want to stress that such an ad-hoc approach is only suitable for well aligned data. Misalignment would increase the variability, making it difficult to discriminate differential frequencies.

## Results and Discussion

The evaluation of the workflow proposed in this paper is outlined in three sections. First, we compare the alignment method against other available methods. Second, we evaluate the quantitative power of the approach. Third, we discuss the efficiency of the workflow.

### Spectral alignment evaluation

We compared the proposed method to two alternatives that use FFT cross correlation to measure correlation: Icoshift [8] and RAFFT [3], The comparison was made using two different datasets: 1) The wine dataset, which is a known benchmark NMR dataset [8]; 2) A Huntington dataset [22]. Details regarding the data and the methodology are outlined in the methods section at the end of this paper, and in the original papers that describe the data [8,22].

**Figure 4:**
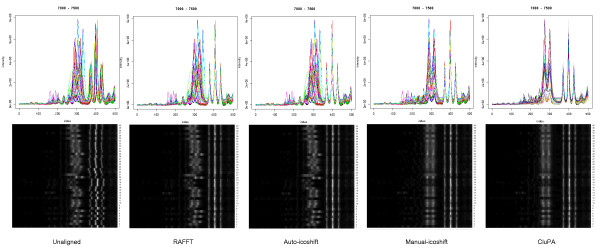
**Spectral plots and grey scale plots of the region 7000-7500 of the Wine dataset**. The spectrum region before and after alignment by different methods (indicated at the bottom) was visualised as a spectral overlay (top) (multiple spectra overlaid in specific color for each spectrum) and as a two-dimensional grey-scale plot (bottom) (x axis: resonance frequency, y axis: spectrum index, lighter zones correspond to peaks).

As mentioned in [8], most methods such as COW, RSPA and Icoshift perform well on certain regions of the spectra, but perform less in specific regions, such as the lactate and ethanol region. Figure 4 shows gray scale representations and spectrum plots of the unaligned spectra and the results of the different alignment methods within this region. RAFFT did not properly align this region. Icoshift fails to properly align the lactic region in the automatic interval selection mode. However, when we specified the intervals from [8] using the user-defined interval mode, a satisfying alignment was obtained. CluPA aligns well on all main resonance peaks mentioned in [8], without the requirement of user interaction. In the case of the lactic acid region, CluPA yields a good alignment, as long the peak detection algorithm successfully detects the peaks in this region.

The same methods were also applied to the Huntington dataset. We show two types of selected regions: a region containing the highest peaks and the largest region containing strongly overlapping peaks.

a) Region with highest peaks: between 39600 and 40600 or 1.54 to 1.07 ppm

**Figure 5:**
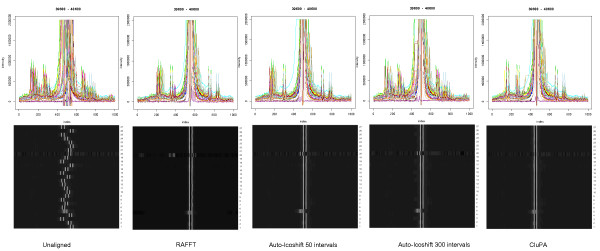
**Spectral plots and grey scale plots of the high-intensity region (39600-40600) of the Huntington dataset**. Visualisation equivalent to Figure 4. The highest intensity reaches approximately to 1.3 * 10^8^. In order to be able to see the smaller peaks, only the lower end of the intensity range was shown in the spectral plots. Due to the high intensity, only the highest peaks are clearly observed in the corresponding grey scale plots.

This region (Figure 5) is also the Lactic acid region, similar to the wine dataset, in which there are many small peaks around a very intense peak. Considering the grey scale plots, RAFFT, Icoshift and CluPA align the highest peak region in an almost identical way. The spectral plots, which boost the lower intensity scales, however show that CluPA generally outperforms the other alignment options with regards to the lower intensity peaks surrounding the highest peaks. Nevertheless there are still some misalignments, potentially caused by the fact that the peak detection algorithm does not fully detect these peaks. This problem may be overcome by adjusting the peak detection parameters. The grey-scale plots also reveal spectra 5 and 20 as commonly misaligned, in accordance to their outlier status (see methods section).

b) Region with high overlap: from 34300 to 35700 index value or from 3.96 until 3.32 ppm

**Figure 6:**
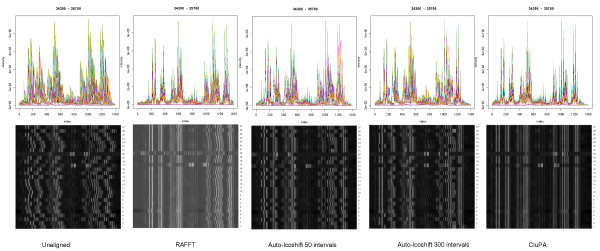
**Spectral plots and grey scale plots of the large and dense region (34300-35700) of the Huntington dataset**. Visualisation equivalent to Figure 4. Prior to alignment, the peaks corresponding to different chemical resonances show significant overlap.

The large number of peaks in this region (Figure 6) makes the alignment of spectra by a binning method challenging, since the bin may contain the wrong peaks for the alignment. Icoshift does give a very good alignment for some spectra, but it is not always successful. In some cases, the peaks are not fully aligned together and there are some artefact lines in the spectral plots of the Icoshift results. Both RAFFT and CluPA show better results in this region. The grey scale pictures show that RAFFT gives a perfect alignment for the left side of the region and yields an adequate improvement in the right side region. The best alignment is obtained by the CluPA algorithm, which correctly matches the high peaks of the region. The grey scale plots also reveals the noisy spectra 5, 17 and 20 as outliers.

**Figure 7:**
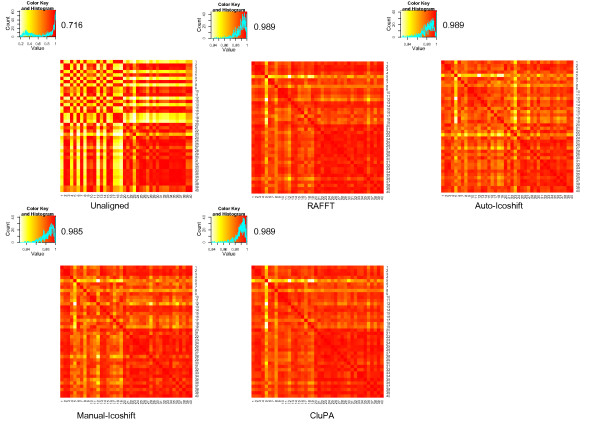
**Pairwise Pearson correlation within the Wine dataset before and after alignment**. Pairwise Pearson correlation coefficients between all spectra, for the different methods. Each dot within the heatmap corresponds to pair of spectra, with colors ranging from white (correlation = 0) to red (correlation = 1). The small inset on the top-left of each map is the histogram showing the distribution of the correlation coefficients and the value in its right side is the average correlation value.

A different way to assess the efficiency of the alignment algorithm consists of a correlation analysis between spectra. To this aim, the Pearson correlation coefficient was calculated between each pair of spectra within the (wine) dataset. A visual representation of all pairwise correlations for each method is shown in Figure 7. In general, all alignment methods yield an apparent improvement. Although Manual-Icoshift works well on all main resonance peaks (as shown on previous figure), its correlation map shows lesser correlation for several spectra. The reason may be found in the fact that Manual-Icoshift does alignment on some user-defined regions of the spectra, and does not fully consider all data points. The correlation maps of RAFFT, Auto-Icoshift and CluPA are similar.

**Figure 8:**
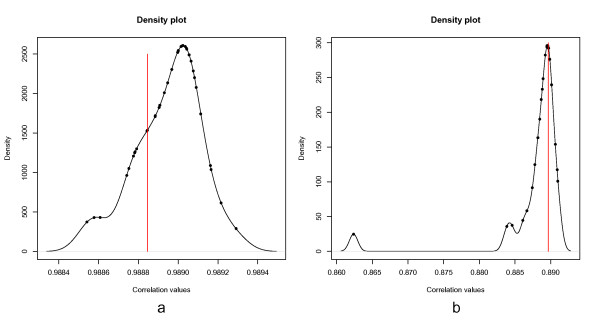
**Distribution of the average correlation values when using different references**. a) The case of the wine data. b) The case of Huntington data. The red lines indicate the correlation values of the references found by the proposed heuristic.

We proposed a heuristic method to find the reference spectrum where other spectra are aligned against. The method selects the spectrum with the maximum goodness, which is computed from the distance between the peaks of the reference candidate and the others. To further evaluate the impact of reference selection, we used a simple test based on the average correlation values, obtained as explained above. Each sample was selected once as reference, and the others were aligned against it through the CluPA alignment algorithm. The distribution of the average correlation values for each run was drawn, and the correlation value corresponding to the use of the reference found by the heuristic method was marked by red lines, in Figure 8.a and 8.b. In the case of the wine dataset, the correlations are high with a narrow distribution. The reference is close to the center of this distribution. In the case of the Huntington dataset, the heuristic selects a reference in the center of the distribution of the non-outliers, clearly demonstrating that the heuristic method works. This is further confirmed by the fact that this heuristic ranks the outlier spectra (5, 17 and 20, see methods section) lowest among all candidate reference spectra (results not shown).

### Quantitative analysis

**Figure 9:**
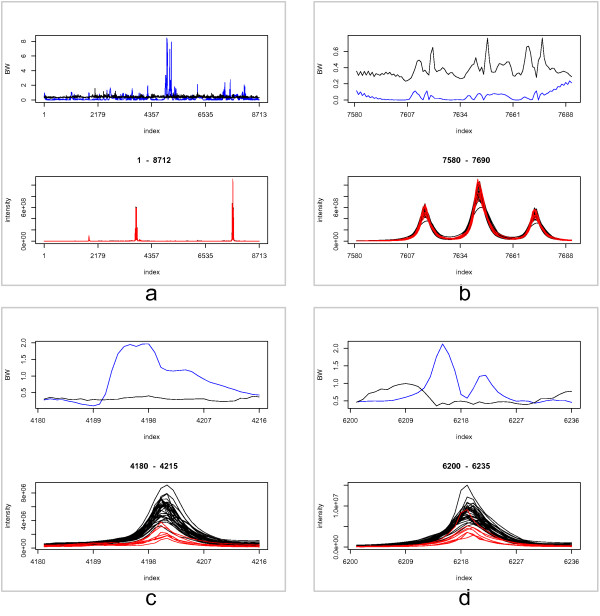
**Quantitative analysis for the wine dataset**. a) top panel: The blue line represents the BW-statistic for the red and white wine dataset. The black line indicates the critical BW-value for rejecting the null hypothesis (i.e., no difference between the groups) at a Bonferonni adjusted alpha-level. The null hypothesis can be rejected when the blue line surpasses the black line. bottom panel: Intensity values of the frequency instances. Black lines indicate the red wine group. Red lines indicate the white wine group. b) close-up in region 7580-7690. c) close-up in the region 4180-4215. d) close-up in the region 6200-6235.

The quantitative analysis was performed as outlined in the workflow section. The alpha-level of 0.05 was corrected for the 1411 peaks found above an intensity of 50,000. The analysis returned 20 regions of interest, which had a significant BW-ratio. The top panel of Figure 9.a displays the significant regions over the entire x-axis. The blue line indicates the BW-statistic and the black line denotes the BW-statistic at a percentile of alpha/1411 using the null distribution. The null distribution is composed out of 1000 samples. BW-values above the black line indicate a differential region. The plot in the bottom panel displays the NMR-spectra for the red and white wine. From the figures it becomes clear that differential regions do not necessarily correspond to regions with intense peak abundances because the BW-statistic takes into account the variability between the groups instead of looking at absolute differences.

In order to demonstrate the proper functioning of the approach, we present several close-ups of interesting regions. For example, Figure 9.b presents a close-up of the most intense peaks in the data (bottom panel). From the top panel it is clear that this region is not selected as a differential region; the BW-statistics stays under the critical value corresponding to the significance level. A different scenario is observed in Figure 9.c and 9.d, where differential regions are detected. A remarkable observation is that in Figure 9.c and 9.d the maxmimum BW-statistic is not reached at the apex of the NMR-peaks but in the ascending and descending shoulder of the peak. It seems that the due to lower variability in the shoulder, i.e., more robust measurements, it should be easier to detect differences. An approach which focuses on finding differences in the peak heights might discard valuable information.

We compared these results with the information in the original research paper [23], and found that most findings are in accordance to the published results. In figure 9.b, the highest intensity region or the ethanol region ranging 1.10-1.25 ppm is not a good region for classification because the authors had to use a very highly complex iPLS model with 6 components for it. Leave one out for RMSECV(Root Mean Squard Error of Cross-Validation) and R2 (Squared correlation cofficient) are 0.31 and 84%, however, the authors reported that they were overfitting due to the complex model [23]. Figure 9.c is the methanol region ranging 3.3-3.4 ppm. The result from the paper showed that they used simpler iPLS model with 4 components, achieve 0.03 and 75% for RMSECV and R2, respectively. This means that this region is suitable for classification (see [23] for more details). We do not have quantification information corresponding to figure 9.d, or the acid acic region.

### Algorithm speed and parameter settings

Since the alignment algorithm (CluPA) requires most of the computational resources of the workflow, in comparison with the statistical analysis, we briefly discuss its operational characteristics in this section. On a Macbook Pro 2,66 GHz (using R package version 2.11.1.) the whole process needs minutes to complete. As other peak based alignment methods, the peak detection process is the most time consuming. The alignment of the Wine dataset takes totally 2.4 minutes, consisting of 1.9 minutes for peak detection, 0.06 minutes for finding the reference and 0.5 minutes for the alignment process. The Huntington dataset required nearly four times the time for the Wine dataset: 9.5 minutes. Most of the time cost is reserved for peak detection (8.0 minutes). The remainder includes 0.04 minutes for finding the reference spectrum and 1.5 minutes for the alignment.

Besides the parameters for the peak detection, the workflow does not require parameters for the initialization prior to running. One extra optional parameter for CluPA, but also for both RAFFT and Icoshift, is the maximum shift point, which is inherited from the FFT cross-correlation function. This value is usually 0.05 ppm, more or less 100 points, depending on the data. If this parameter is not set, the FFT cross-correlation function will check on whole spectral regions to get the optimal shift step. Furthermore, users need to set the parameters for the peak detection algorithm or use other methods instead. Even though default values are set in the software implementation, they are not guaranteed to work optimally for any dataset. The selection of optimal parameters for peak detection algorithm is beyond the scope of this paper.

## Conclusions

In this paper, we proposed a simple and efficient workflow which is centered around recursive hierarchical clustering. This strategy, which is referred to as *speaq *("spectrum alignment and quantitation") is feasible because prior to the alignment, a peak-picking and reference selection approach is used to reduce the complexity related to alignment. The method aligns the target spectrum to the reference spectrum in a top-down fashion and makes use of Fast Fourier Transformation (FFT) cross-correlation to reduce the computation time to find the shift step. As conventional recursive strategy like RSPA or RAFFT, the CluPA algorithm aligns a larger segment and then recursively divides it into smaller segments to refine the alignment. The differences between them lie in the method and criteria to divide the spectra. RAFFT selects the dividing point based on the optimal shifts computed from Fast Fourier Transform Cross-Correlation, RSPA detects in the reference and target spectrum lists of segments, then tries to find the corresponding segments between them, and goes into segments for further alignment. Our proposed method builds a hierarchical cluster tree from peak lists of reference and target, and divides the spectra into smaller regions based on the most distant clusters of the tree. However, instead of considering many clusters simultaneously, we cut off the dendrogram into just two sub trees (corresponding to two clusters) and then go further into each of them for finer alignment. Another feature of the workflow which is different from most other peak-picking based alignment methods is that instead of grouping peaks together to create segments for alignment, *speaq *does the alignment first, then it groups peaks and creates smaller segments. In general, this strategy decreases the shift of peaks before the hierarchical cluster tree is built, thus, it significantly reduces overlapping peaks that yield wrong clustering.

Despite the heuristic approach, selecting the perfect reference remains a non-trivial task, since one spectrum may be a good reference for a specific region but not for other regions. Within the software implementation, we therefore also allow users to intervene into the process by setting reference in the region they are confident about. This also allows to skip certain regions during the alignment. The maximum shift can also be set in a region-specific manner. All this information can be readily provided to the software in an extra text file.

The *speaq *workflow was compared to two widely used methods Icoshift and RAFFT. The method shows several advantages over the others. It is easy to use because users do not need to set many initial parameters. Experiments on both a public dataset and an Huntington dataset show that it performs very well compared to the others. All method use FFT cross correlation for finding the shift because of its advantage in computational time. However, *speaq *is slower than both Icoshift and RAFFT, due to the time for peak detection and its recursive nature, but this is generally not a problem with the powerful computers we have nowadays.

In summary, the *speaq *workflow is unique in the way the subsequent steps are ordered. Because of rearranging the order of processing steps, simple and robust methods could be adopted for the workflow. A case in point is the effective analysis based on the BW-ratio. This could only be achieved since NMR spectra are well aligned. If this were not the case, misalignment would introduce sources of variability into the statistic making it difficult to select differential regions. I should be noticed that the proposed method is still open for improvements. For example, from the top panel of Figure 9.a it can be seen that the null hypothesis (black line) is remarkably constant. This constant line might suggest that the properties of the null distribution are applicable over the entire frequency range. Parametrization of the method can avoid the sampling of the null distribution and improve computational efficiency. The method currently does not take into account covariation of the intensities of peaks of the same molecule across samples [24]. The method was tested only on NMR datasets. Similar data types, such as chromatography data, may also benefit from the approach presented here.

## Methods

### Data sets

#### Wine data

The wine data from [23] includes the 1H NMR spectra of 40 table wines of different origin and color (red, white and rosé) with 8713 data points from 6.00 to 0.50 ppm. The dataset was aligned and analyzed by three different methods. First, Icoshift was used under two parameter settings, namely, auto-Icoshift and manual-Icoshift. The former setting divides the dataset into equally sized intervals. In the latter setting, the dataset is split into pre-defined intervals, which are defined in the spectra according to the knowledge of the user, prior to alignment [8]. Both settings use an average spectrum as reference. Second, RAFFT was applied with the automatically found reference. Third, the *speaq *workflow, described in this paper, was applied on peaks with an intensity larger than 50,000. The reference was automatically found as described.

#### Huntington data

To further demonstrate the quality of our method, a Huntington data set was processed with the proposed workflow. The metabolomics data set [22] combines 1H NMR spectroscopy and chemometrics in order to identify biomarkers in transgenic presymptomatic rats for Huntington disease. It is worth mentioning that there are a few outliers in this dataset: spectra 17 and 20 contain very few and mainly low-intensity peaks, whereas spectrum 5 shows different peak shapes. These irregularities can be attributed to experimental artefacts, but are intentionally not removed in order to assess their impact on the stability of the results.

The data has a large region from 34000 (4.10 ppm) to 36000 (3.18 ppm) wherein different peaks strongly overlap with each other. This makes it a challenge to select suitable regular intervals for Icoshift, as it is easy to divide peaks into wrong intervals. In Icoshift, this problem can be solved by letting the user select the intervals manually. However, even if the user does this, in order to get a more clear separation of small peak regions, these small regions should be considered to be refined after the alignment of the large region, in a way equivalent to recursive alignment algorithms like RAFFT, RSPA and CluPA. As in this dataset, the prior knowledge of the intervals is considered to be unknown, only the automatic version of Icoshift was applied, with a different number of intervals, i.e. 50 and 300. These values correspond to low and high-resolution cases, respectively. RAFFT was applied with the reference found by the proposed algorithm. For CluPA, all detected peaks with intensity less than 50,000 were removed in order to reduce noise-related peaks. The reference was automatically detected as described above, prior to alignment.

## Software availability

A software implementation of the workflow described in this paper is freely available under an Apache 2 license. Source code and documentation can be downloaded from http://code.google.com/p/speaq/.

## Authors' contributions

TNV, KL and DV developed the workflow and methods and wrote the manuscript. The software was implemented by TNV and DV. KS analysed the algorithmic complexity. KV and RD provided and interpreted the NMR data. The computational aspects of this work were supervised by BG and AV, whereas the chemical aspects were under supervision of FL. All authors read and approved the final manuscript.
